# Data on industrial new orders for the euro area

**DOI:** 10.1016/j.dib.2016.08.068

**Published:** 2016-09-10

**Authors:** Gabe J. de Bondt, Heinz C. Dieden, Sona Muzikarova, Iskra Pavlova

**Affiliations:** European Central Bank, Frankfurt am Main, Germany

**Keywords:** Economics, Industrial new orders, Leading indicators, Euro area

## Abstract

This data article provides time series on euro area industrial new orders and is related to the research article entitled “Modelling industrial new orders” (G.J. de Bondt, H.C. Dieden, S. Muzikarova, I. Vincze, 2014b) [3]. The data are in index format with a fixed base year (currently 2010) for total new orders as well as a number of breakdowns. The euro area data are based on the official national data for countries that still collect data and on European Central Bank (ECB) model estimates for countries that discontinued the data collection. The weighting scheme to calculate euro area aggregates makes use of country weights derived from industrial turnover statistics as published by Eurostat.

**Specifications Table**

**Table:** 

Subject area	*Economics*
More specific subject area	*Macroeconomic statistics for conjunctural analysis*
Type of data	*Tables*
How data was acquired	*The euro area series contain the discontinued data by Eurostat (until March 2012) and the model estimates calculated by the ECB for the periods thereafter.*
Data format	*Processed*
Experimental factors	*Hybrid combination of official national data on industrial new orders and model estimates for those countries that have discontinued the data collection*
Experimental features	*Model estimates for industrial new orders are based on a consistent modelling framework using a mix of information sources, i.e. hard and soft data*
Data source location	*Germany*
Data accessibility	*Data are available within this article*

**Value of the data**

• The data fill the existing data gap of euro area industrial new order statistics. Monthly updates provide timely information for users.
• Industrial new orders are used as leading indicator for economic activity (e.g. industrial production, GDP) as well as for analysis and monitoring of economic developments.
• The indicators for new orders are combined with the formerly “official” series on new orders from Eurostat so that users have access to long time series, an important feature for modeling.

## 1 Data

The data on euro area industrial new orders are available in time series format with a fixed base year (2010) in seasonally and calendar adjusted format. The first two columns of Table 1 report the series codes in the ECB׳s Statistical Data Warehouse (SDW) and the starting date, respectively, with the latter providing regular updates at monthly frequency. Besides the total of industrial new orders and total excluding orders for heavy transport equipment, breakdowns are available (i) for the “Main Industrial Groupings” (capital goods, intermediate goods and durable/non-durable consumer goods), and (ii) by origin of demand: domestic and non-domestic (further divided into euro area and non-euro area).

## 2 Experimental design, materials and methods

For those euro area member states that continue with the data collection (e.g. Germany, Italy, and Spain), national new order data are used as input. For the remaining euro area countries (e.g. France, the Netherlands, and Belgium), estimates are derived, based on various model determinants. The detailed model specifications are available in [1], Section 2.3. The Appendices in [1], [2] report detailed country model estimates. The model estimates have yielded solid out-of-sample (see Fig. 4 in [3]) and in real time (see Section 5 in [3]) results.

Data on euro area new orders are subject to revisions in real time, like other economic series, i.e. hard data statistics. Table 2 shows that total new orders excluding heavy transport equipment is comparatively less subject to revisions and among the breakdowns orders for intermediate goods.

## Figures and Tables

**Table 1:** ECB indicators on euro area industrial new orders (annual percentage changes, unless otherwise stated).

								month-on-month		
**Series code**	**Series starts**	**Industrial new orders breakdown**	**2014**	**2015**	**2016 Jan.**	**2016 Feb.**	**2016 Mar.**	**2016 Jan.**	**2016 Feb.**	**2016 Mar.**
STS.M.I8.Y.ORDT.NSC002.3.000	Jan.1995	Total	3.2	2.6	1.2	1.3	−0.6	−0.6	0.0	−0.8
STS.M.I8.Y.ORDT.NSC003.3.000	Jan.1996	Total excluding heavy transport equipment	2.8	2.4	0.3	0.5	−0.2	−0.9	0.0	−0.7

		*By main industrial groupings*								

STS.M.I8.Y.ORDT.NS0042.3.000	Jan.1996	Intermediate goods	1.2	−0.2	−1.2	−1.1	−3.0	−0.6	−0.2	−1.0
STS.M.I8.Y.ORDT.NS0052.3.000	Jan.1995	Capital goods	5.6	7.4	3.9	5.2	5.0	−0.1	1.0	0.8
STS.M.I8.Y.ORDT.NS0082.3.000	Jan.2000	Consumer goods	3.0	3.3	6.7	0.8	3.2	1.8	−3.4	0.2
STS.M.I8.Y.ORDT.NS0062.3.000	Jan.1995	Durable consumer goods	2.8	4.1	5.0	2.5	3.5	2.0	−2.6	−0.5
STS.M.I8.Y.ORDT.NS0072.3.000	Jan.2000	Non-durable consumer goods	3.2	3.7	6.8	1.3	3.4	1.7	−3.0	−0.2

		*By origin of demand*								

STS.M.I8.Y.ORDD.NSC002.3.000	Jan.1998	Domestic	1.4	1.9	−0.2	1.0	−2.3	−1.2	0.6	−1.1
STS.M.I8.Y.ORDE.NSC002.3.000	Jan.1995	Non-domestic	4.4	2.9	2.5	1.1	1.1	0.3	−0.8	−0.4
STS.M.I8.Y.ORDZ.NSC002.3.000	Jan.2003	Euro area	4.4	5.6	4.1	4.0	2.5	−1.2	0.2	0.1
STS.M.I8.Y.ORDX.NSC002.3.000	Jan.2003	Non-euro area	5.4	5.0	5.0	3.1	4.2	0.9	−1.9	1.1

**Table 2:** Historic revisions in the month-on-month growth rates of the ECB indicators on euro area industrial new orders (*May 2012 to February 2016*).

**Industrial new orders breakdown**	**Average**	**Average absolute**	**Range of revisions**^a^	
	**revision**^a^	**revision**^a^	**Total**	**90%**
Total	0.02	0.46	−1.5 to 1.7	−1.0 to 0.9
Total excluding heavy transport equipment	0.04	0.39	−1.2 to 1.1	−0.8 to 0.7

*By main industrial groupings*				
Intermediate goods	−0.03	0.45	−1.3 to 1.4	−0.8 to 1.0
Capital goods	0.09	0.85	−3.5 to 2.3	−2.8 to 2.1
Consumer goods	−0.02	0.80	−2.7 to 2.2	−2.0 to 1.8

Durable consumer goods	−0.07	1.07	−2.9 to 3.3	−2.1 to 2.2
Non-durable consumer goods	−0.03	0.83	−3.4 to 2.3	−1.6 to 2.0

*By origin of demand*				
Domestic	0.14	0.58	−1.3 to 2.4	−1.0 to 1.7
Non-domestic	−0.08	0.65	−1.7 to 1.5	−1.6 to 1.0
Euro area	−0.08	0.74	−3.5 to 3.7	−1.3 to 1.7
Non-euro area	−0.22	0.70	−2.2 to 1.5	−1.9 to 1.0

a Average revision: differences between the latest (March 2016 release) and first release divided by the number of observations. Average absolute revision: absolute differences between latest and first release divided by the number of observations. Range of revisions: highest and lowest values of all / 90% of the revisions.
